# Cross-reactivity of sIgE to mite and shrimp induced allergies in different age groups and clinical profiles of shrimp sIgE in vegetarians

**DOI:** 10.1038/s41598-019-49068-2

**Published:** 2019-08-29

**Authors:** Cheng-Ying Shen, Jaw-Ji Tsai, En-Chih Liao

**Affiliations:** 1 Department of Medicine, Mackay Medical College, New Taipei City, Taiwan, ROC; 2 Division of Allergy, Immunology & Rheumatology, Department of Internal Medicine, Asia University Hospital, Taichung, Taiwan, ROC; 3 Division of Allergy, Immunology & Rheumatology, Taichung Veterans General Hospital, Taichung, Taiwan, ROC; 4 College of Life Sciences, National Chung Hsing University, Taichung, Taiwan, ROC

**Keywords:** Immunology, Immunology, Allergy, Allergy

## Abstract

The sensitization to house dust mites (HDMs) and shrimps affects the development of hypersensitivity with an increase in age. Due to the cross-reactivity between shellfish and HDMs, HDMs were considered as the primary sensitizer for shellfish allergy. Thus, vegetarians might be sensitized to shrimp through the inadvertent inhalation of HDMs. Therefore, we assessed the prevalence of shrimp or mite allergy among different age groups and vegetarians. The serum specific-IgE (sIgE) level of HDMs and shrimp in 60 children/adolescence (un-adults), 30 adults, 30 elderly, and four vegetarian adults patients were measured. The sera with sIgE levels greater than 3.5 kUA/L were cross-reactivity examined. We found that HDMs induced higher sIgE than shrimp in un-adults. In contrast, shrimp-induced sIgE was higher in the adults and elderly patients. Moreover, adults were more frequently sensitized to shrimp and mite at the same time compared with the un-adult or elderly groups. The mite-Der p 10 not only displayed high cross-reactivity to the shrimp-Pen a 1 in all age groups and vegetarians but functioned as the major allergen to sensitize un-adults. Overall, the level of mite or shrimp sIgE is influenced by alterations in age, and vegetarians are at risk of shrimp sensitization via cross-reactivity between shrimp and mite.

## Introduction

Increasing evidence demonstrates that the sensitization to indoor allergens is a causative factor for the development of airway hypersensitivity^1,2^. In the tropical and subtropical zones, the major indoor allergens are HDMs, such as *Dermatophagoides pteronyssinus* (*D. pteronyssinus*)^3,4^. More than 20 IgE-binding allergenic components of *D. pteronyssinus* have been identified. The group 1 and group 2 allergens of *D. pteronyssinus* are identified as major allergens and have been well studied^5,6^. In this study we have focussed on group 10 allergen of *D. pteronyssinus* as they show cross-reactivity to allergens in invertebrates and seafood^7^. With a high homology to tropomyosin in seafood, Der p 10 has been described as a prominent challenger for severe systemic anaphylaxis^8,9^.

In addition to allergies from HDMs, food allergies have also become an increasingly troublesome issue over the last few decades. Food allergens can induce symptoms, such as rhinitis, urticaria, and anaphylaxis in patients^10^. The prevalence and types of food allergy vary according to age, local diet, and genes, and are triggered by various allergens sources, such as milk, eggs, peanuts, fish, and shellfish^11,12^. Shellfish, including crustaceans and molluscs, cause the most food allergies in both children and adults worldwide. Among shellfish, shrimp is most involved in allergic reactions, as it contains tropomyosin. It has been reported that with high sequence homology to shrimp tropomyosins, HDM allergens might be the primary sensitizer for shrimp allergy via cross-reactivity^13,14^. A previous study indicated that mite-specific IgE might be a risk factor for shrimp allergy^15^. Therefore, the clinical relevance of sensitization to the allergenic components of daily diets must be taken into consideration.

An increasing number of people decided to choose a vegetarian diet due to several reasons such as ecological and religious. A vegetarian diet may also play a beneficial role in promoting health and preventing food allergy. Previous studies indicated that the consumption of vegetables and fruits could increase antioxidants to combat inflammation, which also induces anti-asthmatic effects^16,17^. However, it is a controversial subject as other studies have reported that allergens derived from nuts, fruits, and vegetables can induce food allergies in adolescents and adults^18–20^. Moreover, vegetarians might suffer anaphylaxis via inhaled allergens from HDMs inadvertently^21,22^.

Children are the most common population who are affected by respiratory allergies caused due to the exposure to indoor aeroallergens^23^. Although sensitization to HDMs is known to occur mostly during the early years of life via exposure to HDMs allergen^24^, the repeated exposure of other allergens changes the sIgE sensitization in the development of symptomatic allergic disease as people get older^25^. Therefore, the aim of this study was to investigate the prevalence and severity of shrimp or mite allergies among different age populations and vegetarians. Moreover, to validate the importance of cross-reactivity among environmental allergens, four vegetarians in the adult group who were not exposed to shrimp allergens were also analysed. Through our study, we hope to clarify the allergen levels induced by mite and shrimp in different age groups and making changes in the dietary habits to prevent the immune response due to consumption of improper food.

## Results

### The sIgE level of patients in different age groups with single allergic sensitization

**Figure 1:**
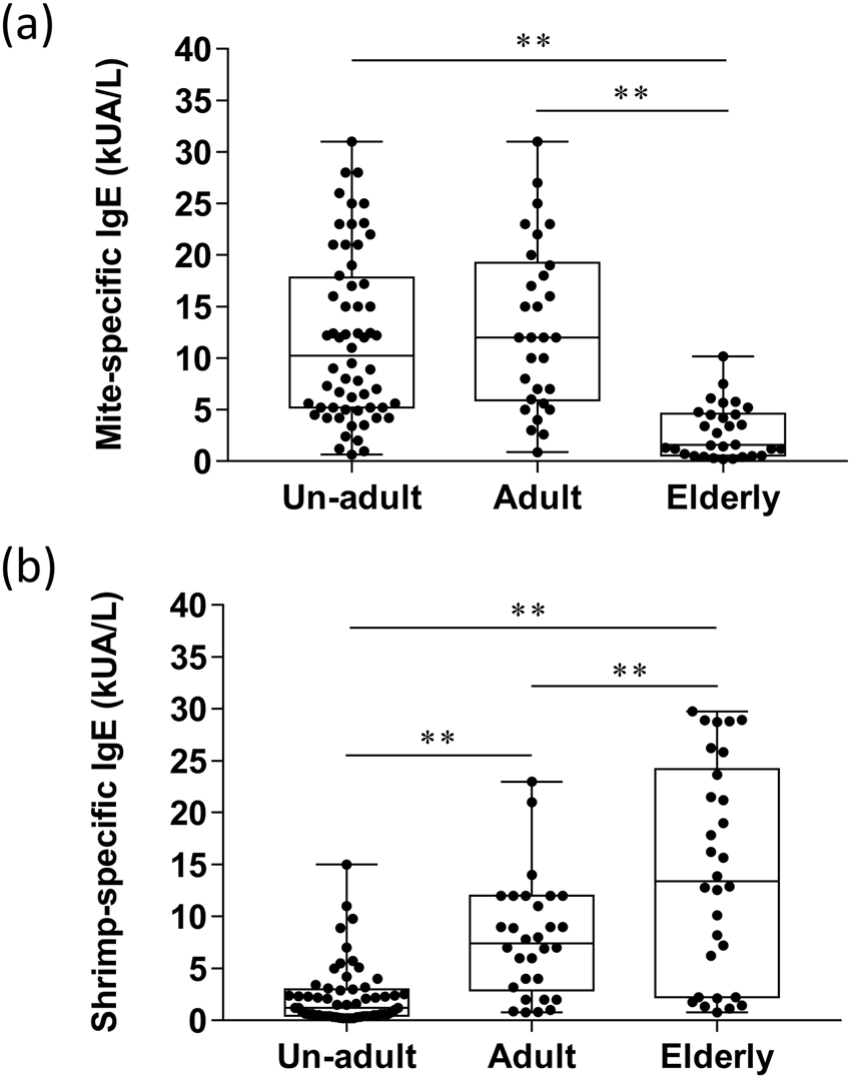
The IgE level of mite or shrimp in un-adults, adults, and elderly. The mite- or shrimp-specific IgE level was determined with ImmunoCAP system. The results were displayed as a box-plot diagram showing the different age groups (X-axis) and specific IgE level in kUA/L (Y-axis). (**a**) The sIgE level of mite in different age groups. **P < 0.01. (**b**) The sIgE expression level of shrimp in these three groups. **P < 0.01, unadult group (n = 60), adult group (n = 30), and elderly group (n = 30). Data were analyzed by one-way ANOVA with Tukey’s multiple comparation post-test.”

A total of 120 allergic outpatients aged between 3 to 80 years was recruited in this study. The results of the association between age and the mite sensitivity showed that the average levels of *D. pteronyssinus* sIgE were 11.9 kAU/L, 13.1 kAU/L, and 2.8 kAU/L in un-adults, adults and the elderly, respectively. The average level of mite sIgE in all allergic subjects who were young, or adults was approximately 10% greater than that in the elderly (Fig. 1a). The average levels of shrimp sIgE were 2.4 kAU/L, 7.8 kAU/L, and 14.3 kAU/L in un-adult, adults and elderly groups, respectively, thus indicating a significant increase in IgE reactivity to shrimp along with increasing age (Fig. 1b).

**Figure 2:**
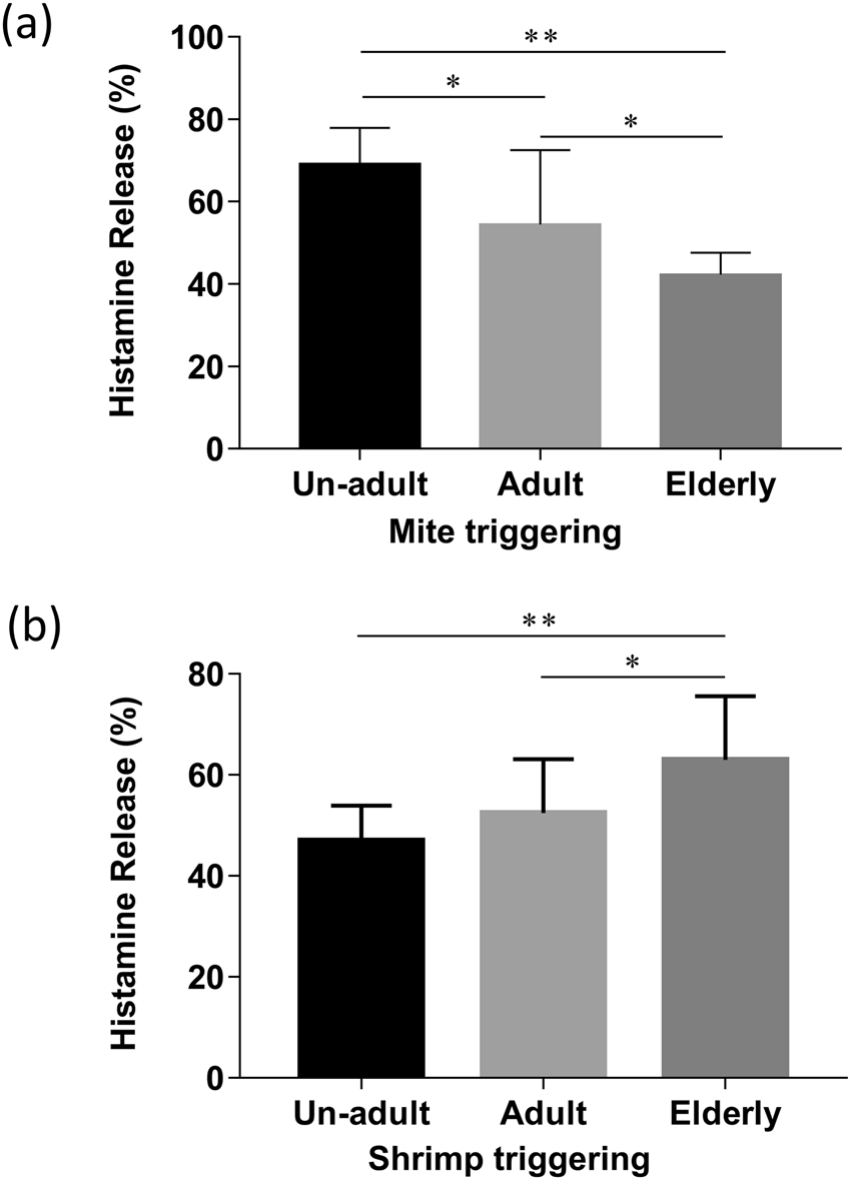
The sIgE induced histamine release in un-adults, adults, and elderly. The histamines releases induced by sIgE of both mite and shrimp (greater than 3.5 kUA/L) in sera were determined with BHR assay. The results were displayed as histogram with standard deviation showing the different age groups (x-axes) and percentage of histamine releases after mite or shrimp triggering (y-axes). (**a**) The percentage of mite sIgE induced histamine release in different age groups. *P < 0.05, **P < 0.01. (**b**) The percentage of shrimp sIgE triggered histamine release in different age groups. *P < 0.05, **P < 0.01, n = 10 in un-adult group, n = 15 in adult group, and n = 11 in elderly group. Data analysed by one-way ANOVA with Tukey’s multiple comparation post-test.

The patient’s serum having high levels of both mite and shrimp sIgE (greater than 3.5kUA/L) were used to validate the age-related sensitization between mite and shrimp. The results of BHR assay showed that the percentages of histamine release were 69.27% in un-adults, 46.96% in un-adults, and 42.29% in elderly after stimulating with mite allergen (Fig. 2a). However, the mean of histamine release was 46.96% in un-adults, 52.40% in young adults, and 62.96% in elderly after treating with shrimp allergen. (Fig. 2b).

### The severities of both mite and shrimp induced allergy in the same age groups

**Figure 3:**
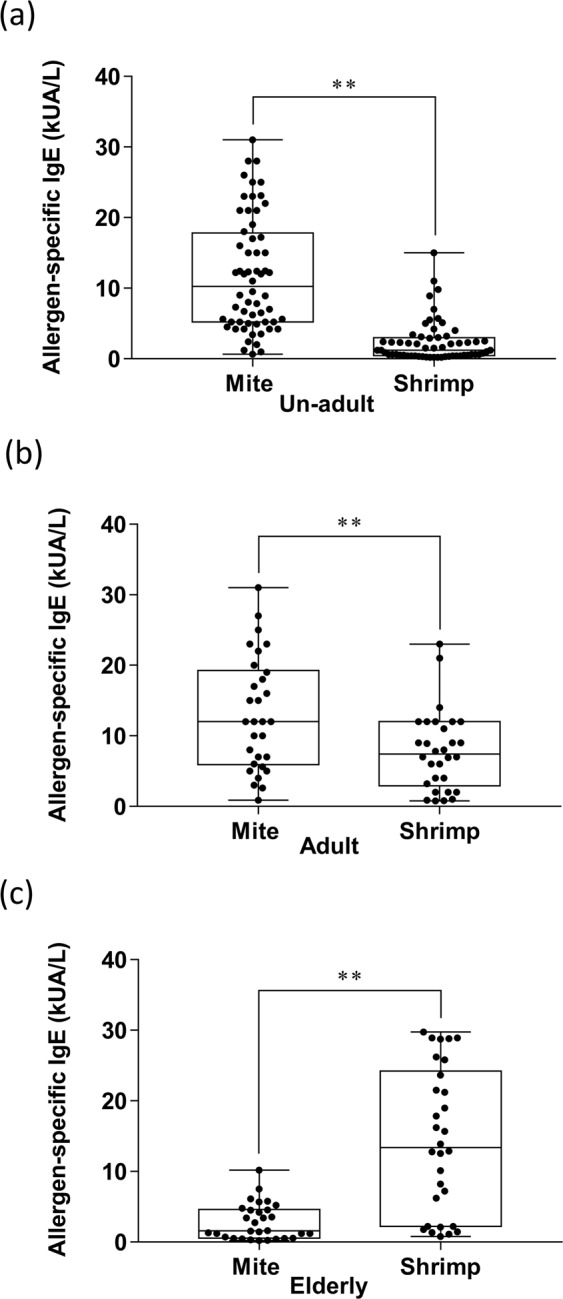
The comparison of mite- and shrimp-specific IgE level in patients in the same age group. (**a**) The sIgE level of mite and shrimp in the un-adult group. (**b**) The sIgE level of mite and shrimp in the adult group. (**c**) The sIgE level of mite and shrimp in the elderly group. Data were analyzed with Student’s *t*-test. **P < 0.01, n = 60 in un-adult group, n = 30 in adult group, and n = 30 in elderly group.

To further validate whether different degrees of sensitization to mite or shrimp is age-related, the sIgE level to both mite and shrimp in each of the three groups were analysed. The results showed that the un-adult (Fig. 3a) and adult (Fig. 3b) groups had higher mite-sIgE levels (11.9 kUA/L and 13.9 kUA/L, approximately) as compare to the shrimp-sIgE levels (2.4 kUA/L and 8.3 kUA/L, approximately). However, the elderly group had higher sIgE levels against shrimp (17.5 kUA/L) than mite (3.3 kUA/L). (Fig. 3c). A similar result was found in the BHR assay by using the patient’s serum with mite and shrimp sIgE, which is greater than 3.5 kUA/L. The mite allergen -induced basophil histamine release has increased in un-adults and decreased in the elderly population, respectively when compared with the induction by shrimp allergen (Supplementary Fig. 2a,b). However, the histamine release was not different in mite or shrimp triggering in the adult group (Supplementary Fig. 2c).

### The correlation of sIgE for mite and shrimp in different aged patients with mite or shrimp allergic sensitization

**Figure 4:**
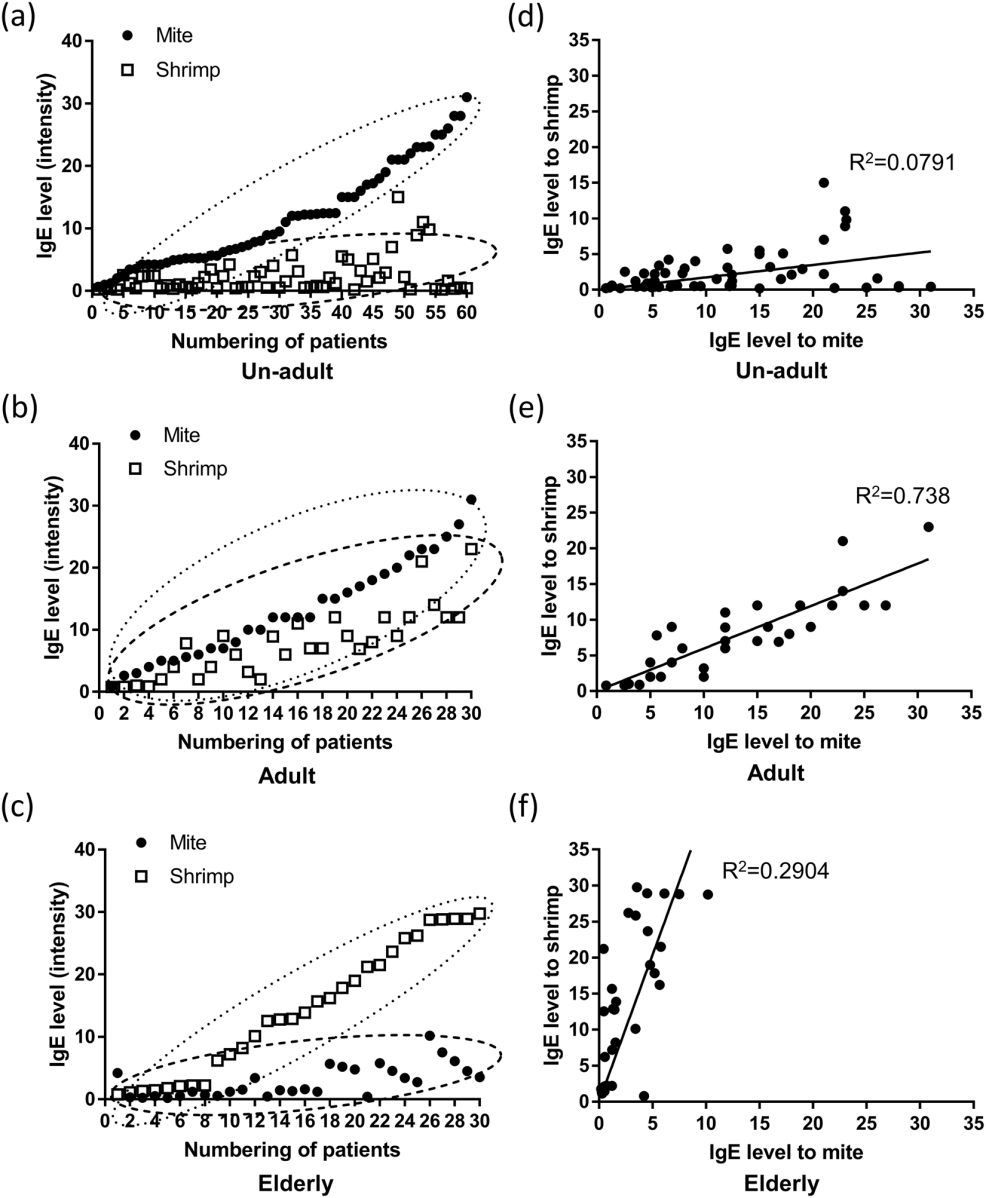
A correlation analysis of mite- and shrimp-specific IgE level in patients of different age groups. The scatter plot of mite and shrimp sIgE in the same individual allergic subjects in the un-adult (**a**), adult (**b**), and elderly (**c**) group. The linear regression analysis in the un-adult group (**d**), adult (**e**), and elderly (**f**) group. (n = 60 in un-adult group, n = 30 in adult group, and n = 30 in elderly group). Black dots: mite group. Hollow square: shrimp group.

The correlation of sIgE expression level in 120 patients was analyzed to examine whether the sensitization to both mite and shrimp in the individuals was age-related. The charts show the different degrees of allergy to mite, indicated by black dots, and to shrimp, indicated by hollow squares, in the same allergic subjects. Most of the individual un-adults show higher levels of sIgE against mite but lower levels of IgE against shrimp (Fig. 4a). The degree of allergy to mite and shrimp was similar in each adult (Fig. 4b). On the contrary, the levels of IgE against shrimp were singly higher among the elderly allergic patients, but those of IgE against mite were lower (Fig. 4c). The results of scatter plots showed that two clusters of sIgE level of mite or shrimp group separated in un-adults and elderly (Fig. 4a,c) but not in the adult group (Fig. 4b). The results of linear regression analysis showed that IgE against *D. pteronyssinus* weakly correlated with shrimps in the un-adults (R^2^ = 0.079) (Fig. 4d) and in elderly groups (R^2^ = 0.29) (Fig. 4e). Tests of serum IgE reactivity showed a significant correlation between shrimp and mite in the adult population (R^2^ = 0.765; p < 0.05) (Fig. 4f).

### Cross-reactivity examination of mite-Der p 10 and shrimp-Pen a 1 in different ages groups sensitized with both allergens

**Figure 5:**
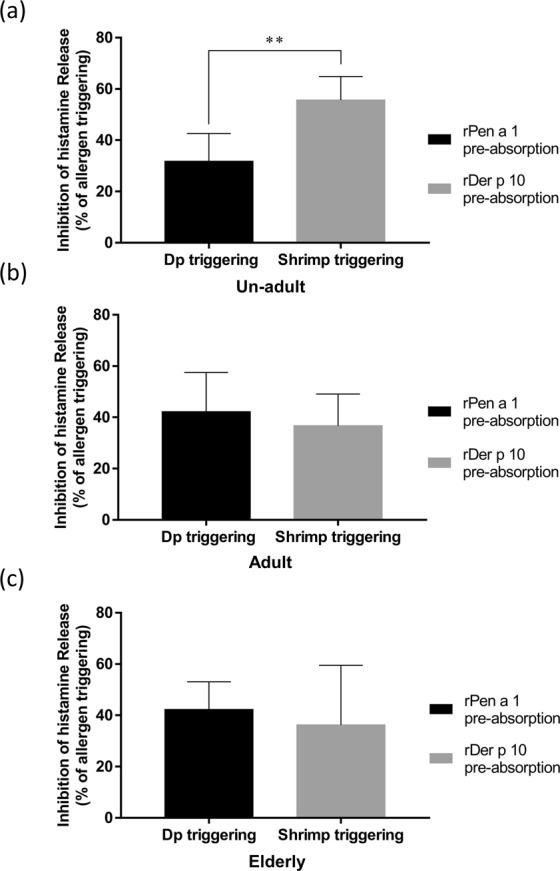
Inhibition in histamine release using rPen a 1 or rDer p 10 pre-absorbed sera from patients in different age groups. The cross-reactivity of rPen a1 and rDer p 10 was examined by inhibition of histamine release. The percentage of inhibition of histamine released from basophils upon treatment with rPen a 1 or rDer p 10 pre-absorbed sera from unadult group (n = 10) (**a**), adult group (n = 15) (**b**), and elderly group (n = 11) (**c**), after triggering allergy with crude extracts of mite or shrimp. Data were analyzed using an unpaired Student’s *t*-test. **P < 0.01.

Due to few studies focused on cross-reactivity of Der p 10 and Pen a 1, sera were used to investigate if mite Der p 10 cross-reacts with shrimp Pen a 1 in double sensitized patients in different age groups. When we compared the groups that showed sensitization to mite (rDer p 10) or shrimp (rPen a 1) allergens without absorption, we observed that in case of absorption, the inhibition of histamine release in sera of un-adults, adult, and elderly by approximately 31.7%, 42.2%, and 42.2%, respectively, in shrimp triggering, whereas the inhibition of histamine release by approximately 55.7%, 36.7%, and 36.2% in un-adults, adult, and elderly, respectively in mite triggering (Fig. 5a–c). Moreover, in the un-adult group, the inhibition of histamine release was significantly higher in shrimp after rDer p 10 absorption than in mite after rPen a 1 absorption (Fig. 5a). On the contrary, there were no significant differences in histamine release due to either mite after rPen a 1 absorption or shrimp after the rDer p 10 absorption in adults (Fig. 5b) and elderly (Fig. 5c).

### Identification of shrimp allergenic components from vegetarians by IgE immunoblot inhibition with Der p 10

**Figure 6:**
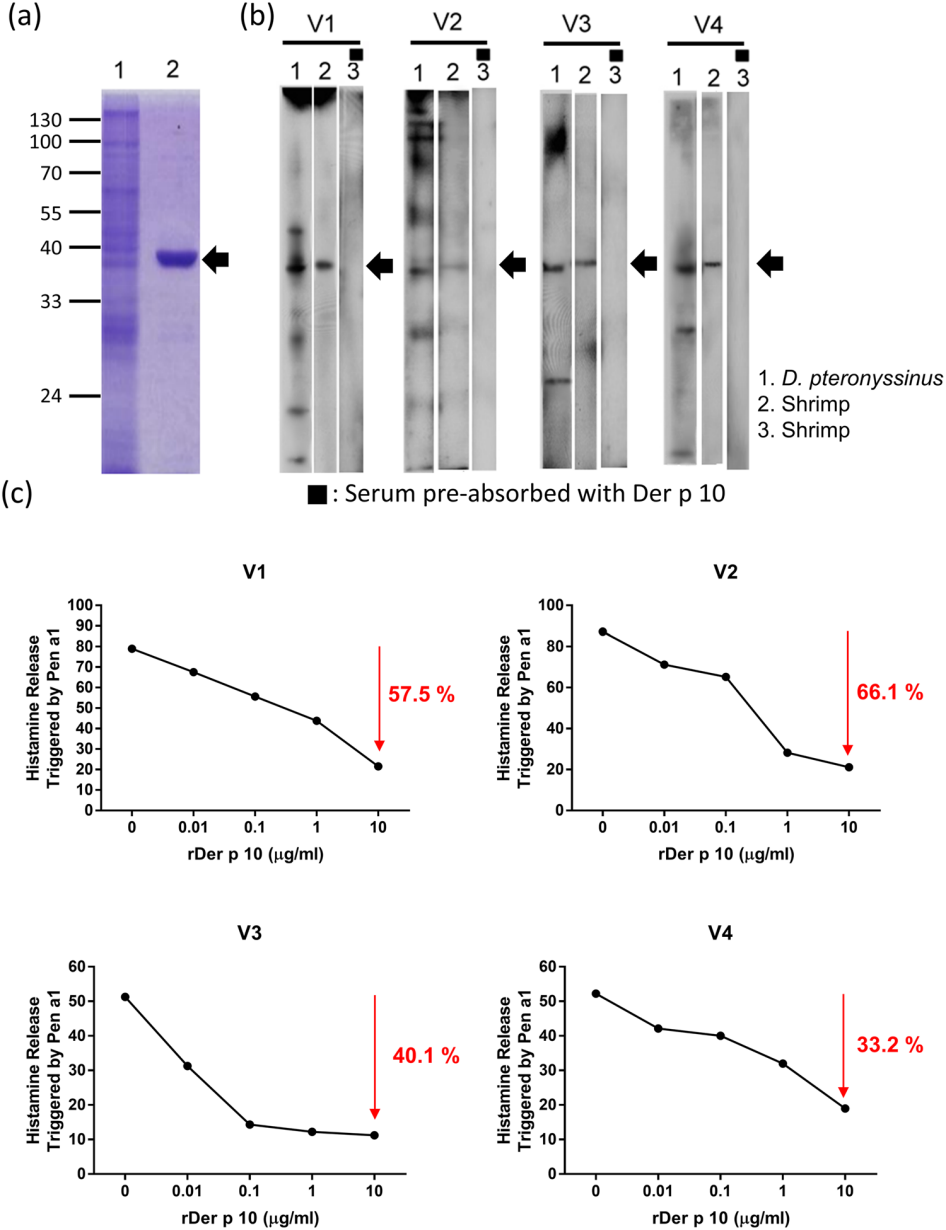
Identification of shrimp allergen in vegetarians by IgE immunoblot inhibition and inhibition of histamine release with rDer p 10. (**a**) Coomassie blue staining of an SDS-polyacrylamide gel loaded with protein samples. Lane 1, the crude extracts of *D. pteronyssinus*. Lane 2, Protein sample of rDer p 10 with a molecular weight of 37 kDa. (**b**) Four sera samples from vegetarian patients (V1-V4) were analyzed by immunoblotting. Lane 1, immunoblot of IgE in sera with crude extract of *D. pteronyssinus* hybridization. Lane 2, immunoblot of IgE in sera with crude extract of shrimp hybridization. Lane 3: immunoblot of IgE in sera with shrimp hybridization pre-treated with 50 μg/ml rDer p 10. Arrows indicate the IgE-binding components with a molecular weight of 37 kDa. The lanes were separated from the same PVDF membranes for the different hybridization conditions. The full blots are presented in the Figure. The grouping of the blots of all lanes was separated with white space. (**c**) The inhibition percentage of histamine release in the vegetarian adult group (n = 4) pre-absorbed with increasing concentration of rDer p 10.

Since our data indicated that rDer p 10 cross-reacted with rPen a 1 (Fig. 4), we hypothesised that the exposure of environmental allergens from mite might induce shrimp-related inflammation in vegetarians. The results of the coomassie blue staining showed that the molecular weight of the rDer p 10 is approximately 37 kDa (Fig. 6a, lane 2, black arrow). The results of immunoblotting for sera of vegetarians showed that all vegetarian subjects possessed the IgE-binding component of *D. pteronyssinus* (Fig. 6b, lane 1) and shrimp (Fig. 6b, lane 2), with a molecular weight of 37 kDa. After the immunoblot inhibition of rDer p 10 absorption, the 37 kDa IgE-binding component could be completely absorbed by rDer p 10 (Fig. 6b, lane 3 black arrow). Furthermore, serum samples from vegetarian subject (V1) were pre-incubated with 0 µg/ml (absence of rDer p 10) to 10 µg/ml of rDer p 10, the histamine release was reduced from 79% to 21.5%. These data suggest that approximately 57.5% of histamine release was reduced by pre-incubation with 10 µg/ml of rDer p 10. The results showing the reduction of histamine release in V2 (66.1%), V3 (40.1%), and V4 (33.2%) are shown in Fig. 6c.

## Discussion

In this study, we found that the allergens from mite can induce severe allergy than from shrimp in most of the allergic subjects with age less than 18 years. On the other hand, un-adults and adults were more sensitized to HDM-induced allergic reaction than elderly patients, while severe allergy induced by shrimp frequently occurred in the elderly population. According to the sIgE expression profile of patients, mite allergen can induce severe allergic reaction than shrimp allergen in the un-adults. However, when compared to the mite-induced allergy, the elderly population suffered dramatical allergy induced by shrimp allergen. Our data of correlation analysis of sIgE for mite and shrimp in different aged patients indicated that there were more notable effects of mite sensitization accompanied with higher IgE against mite in the un-adult group. Moreover, there were co-sensitization and similar IgE responses against mite and shrimp in the adult group. In addition, there were more distinguished effects of shrimp sensitization accompanied with higher IgE against shrimp in the elderly group. The results of cross-reactivity examination showed that mite allergen Der p 10 and the shrimp allergen Pen a 1 displays high cross-reactivity among the three groups, and HDMs play an important role in being the main source of allergens for sensitizing un-adults to shrimp allergens in Taiwan. In addition, our data indicated that the vegetarians still sensitize to shrimp that are related to the cross-reactivity between shrimp and mite.

It is reported that mite and shrimp allergens can elicit strong IgE and T cell responses in patients^26,27^. In this study, we found that children or teenagers younger than 18 years were more sensitive to mite when compared with the adults and elderly. This observation is in line with the hyper-sensitization of HDM allergen in children living in other counties^28–30^. Previous studies had proposed that exposure to HDM allergen (2 μg/g) and dust (10 μg/g) be used as threshold values for the development of allergic sensitization and asthma, respectively^31–33^. Moreover, a nationwide estimate of HDM allergen levels in US bedrooms indicated that the percentages of HDM allergen concentrations at or greater than 2.0 μg/g, and 10.0 μg/g in US beds are 46.2%, and 24.2%, respectively^34^. Therefore, the long-stay at home on the bed with a long exposure of HDMs for most of the un-adults may be the reason for the high percentage of mite-sIgE expression. The presence of HDMs allergen in human breast milk might be a potential inducer to promote allergic sensitization in un-adults^35^.

Although shrimp is a frequent cause of food allergy, the diagnosis of shrimp allergy is often a challenge for clinicians, particularly when allergic patients show either mild symptoms or no consistent reaction on shrimp ingestion. Previous studies indicated that the rate of serious allergic reaction to shrimp was 12% in children and 42% in adults^36^, and to seafood is 3.22% in teenagers and 66.2% in adults^37^. In this study, we addressed that the age-related allergic response was observed in patients with shrimp induced sensitization. Therefore, age is a potential factor for the diagnosis of susceptibility to shrimp-induced allergy. In addition, similar trend of age-related allergic responses was observed in patients with mite induced sensitization in this study.

A previous study indicated that the level of sIgE for shrimp and Der p, which is greater than 3.55 kUA/L and 3.98 kUA/L, respectively, can be used to diagnose shrimp allergy in non-HDM sensitized patients^38^. Our data showed that the correlation of sIgE expression for shrimp and Der p is the highest in adult than in un-adult and elderly allergic patients. Therefore, the high level of sIgE for mite may also be used to pre-diagnose the shrimp allergy and vice versa in adults. In addition, the results of the linear regression assay indicated that adults with mite allergy had a higher risk of being sensitized to shrimp when compared with the un-adult or elderly groups.

The modified cytokine profiles suggested an age-related shift from a Th1 to Th2 response^39,40^. Th2 cells are effective activators of B cells and trigger IgE responses^41,42^. Since boiled shrimp or shrimp derivatives are considered a delicacy in Taiwan, the continually boosted B cells along with accidental ingestion of shrimp derivatives in adults and elderly might be the reason for a higher shrimp sIgE in the elderly population as compared to the adolescent group.

A previous study indicated that the major allergen tropomyosin is also commonly found in cockroaches and HDMs^43^, which contributes to the cross-reactivity among these species^44,45^. Pen a 1 is a major shrimp allergen belonging to the tropomyosin protein family^46^. Comparison of amino acid sequences between Pen a 1 IgE-binding epitope and Der p 10 (Supplementary Fig. 3) indicates that Der p 10 has seven homologous motifs or sequences, spanning between 88–101, 137–141, 144–151, 187–197, 249–259, 266–272, and 273–281, that are aligned with the IgE binding epitopes identified in Pen a 1 IgE epitopes^47^. A previous study indicated that 4 out of 7 homologous sequences (187–197, 249–259, 266–272, and 273–281), located at the N-terminus of Der p 10, showed similar IgE-binding reactivity to sera from shrimp-allergic patients^48^. Based on our results of inhibition of histamine release, we demonstrated that four homologous sequences in Pen a 1 and Der p10 are sufficient to induce cross-reactivity in both mite- and shrimp-sensitized patients.

Environmental factors, lifestyles, and dietary habits play critical roles in allergen sensitization and allergic disease development. According to the results of cross-reactivity in vegetarians, we found that the serum IgE raised against shrimp allergens in vegetarians might be due to prior sensitization to the cross-reactive tropomyosin from mite. This suggests that the tropomyosin in HDMs is a major cross-reactive allergen involved in triggering IgE-mediated hypersensitivity to shrimp. Therefore, vegetarians are also at the risk of hypersensitization to shrimp allergens if previously sensitized by HDM allergens, and they should, therefore, be mindful of their dietary habits.

## Conclusion

In this study, we found that the allergens in HDMs are a leading sensitizer for shrimp allergens in un-adult or adult populations due to cross-reaction. In addition, the potential risk of hypersensitization due to HDM-induced cross-reactivity also exists in vegetarians. Therefore, monitoring diet (vegetarian versus non-vegetarian) and age is a useful way to prevent sensitization to shrimp allergens triggered by the inevitable exposure to cross-reactive HDM allergens.

## Materials and Methods

### Study subjects

The allergic subjects who were diagnosed with at least one of allergic diseases, including allergic asthma, allergic rhinitis, conjunctivitis, atopic dermatitis, and anaphylaxis, and whose sIgE from sera for both mite and shrimp was greater than 0.1 kUA/L (examined by ImmunoCap assay) were selected. A total of 120 allergic subjects were recruited, including 60 children/adolescence (un-adults, <18 years), 30 adults (19–50 years), and 30 elderly (>51 years), who attended the Allergy and Clinical Immunology outpatient clinics at Taichung Veterans General Hospital (TCVGH), Taiwan. The Institutional Review Board of TCVGH reviewed and approved the ethical conduct of this study (IRB No. 940623/C05111). All methods were performed in accordance with the relevant guidelines and regulations of TCVGH. Verbal consent and written informed consent were obtained from the participants after detailed explanations were provided. Blood samples were obtained for further examination. Informed consents of human participants under the age of 18 years were obtained from their parents.

### Determination of IgE antibodies in sera against shrimp and HDMs

Each of the 5 mL blood samples was collected in the serum separator tubes (Kendall, Minnesota, USA) and processed within 4 h. The serum samples were stored at -20 °C until analysis. The allergen-specific IgE was measured by the ImmunoCap^®^ (Phadia CAP no. k77, Uppsala, Sweden) assay using the UniCAP 250 system, following the supplier’s instructions. Blood samples were drawn, and sera were stored for the measurement of IgE specific to HDM (*D. pteronyssinus)* and shrimp- (*Penaeus aztecus)*. The concentrations greater than 0.35 kUA/L and 3.5 kUA/L were considered as positive and high expression, respectively. The assay was automatically performed, and the results were calibrated against the World Health Organization standard for IgE. For linear regression analysis, the patient’s sIgE for both mite and shrimp from un-adults, adults, and elderly were used.

### Preparation of mite and shrimp crude extracts

The crude extracts of mite and shrimp were prepared as described previously^49^. The crude extracts of mite- *D. pteronyssinus* and shrimp- *P. aztecu* were prepared from lyophilized whole-body extracts purchased from Allergon (Allergon AB, Angelholm, Sweden). One gram of frozen mites and shrimps were homogenized by TissueLyser II (QIAGEN, Maryland, USA) in 15 ml phosphate-buffered saline (PBS, pH 7.2). The homogenate was then centrifugated at 14 000 × *g*, and supernatants were collected and stored at -20 °C. Protein concentration was determined by the Bradford assay according to the manufacturer’s instructions (Bio-Rad, California, USA), using bovine serum albumin as a standard.

### Preparation of recombinant allergens of shrimp and mite

The recombinant allergens of *P. aztecus*, (Pen a 1) were purchased from Indoor Biotechnologies (Product Code: NA-STM-1) (Indoor Biotechnologies, Bangalore, India).

The cDNA of Der p 10 was synthesized using primer dT_17_ and MMLV reverse transcriptase (Invitrogen, Massachusetts, USA) from total mRNA of *D. pteronyssinus*. The specific primers of Der p 10 were designed based on the accession number, AF016278.1^50^, provided in the National Center for Biotechnology Information. The polymerase chain reaction (PCR) products of Der p 10 with six histidine (His) were ligated into a pQE30 vector and transformed into *Escherichia. coli*- M15. The selection was performed using kanamycin (25 μg/ml) and ampicillin (100 μg/ml). The 6 × His tagged recombinant proteins were induced using 1 mM isopropyl-β-D- thiogalactopyranoside (Promega, Wisconsin, USA). The proteins were purified by nickel-nitrilotriacetic acid agarose metal affinity column chromatography under native conditions. The results of purified rDer p 10 are shown in the Supplementary Fig. 1.

### Basophil histamine release (BHR) assay

For BHR assay, 10 un-adults, 15 adults, and 11 elderly whose sIgE for both mite and shrimp was greater than 3.5 kUA/L were selected. Firstly, basophils were prepared as follows: Twenty milliliters of whole blood sample were collected from non-atopic donors and carefully layered with 20 ml Polymorphpret^TM^ solution (Axis-Shield, Oslo, Norway) in a 50 ml tube, centrifuged at 500 × *g* for 30 minutes at room temperature. After the centrifugation, the whole blood sample was separated into four layers, including plasma, mononuclear cells, polymorphonuclear cells (PMNs), and red blood cells. The PMNs were harvested and washed with HEPES-buffered saline (0.85% NaCl, 10 mM HEPES-NaOH, pH 7.4), then centrifuged at 400 × *g* for 10 minutes. PMNs were then resuspended in (RPMI)-1640 medium (ThermoFisher, Massachusetts, USA) to a density of 2 × 10^6^ cells/ml. Secondly, detection of the stimulated release of histamine from basophils was performed as follows: basophils were pre-sensitized with sIgE from the allergic subject sera for 4 h at 37 °C. The sensitized cells were triggered using 50 μl/ml crude extracts of *D. pteronyssinus* and Shrimp for 30 min at 37 °C. The supernatant was collected after centrifugation, and was reacted with *O*-phthalaldehyde (OPA, 5 mM) (Sigma-Aldrich Merck, Missouri, USA) for 7 min. The reaction was stopped with 0.04 M sulfuric acid (H_2_SO_4_) (Sigma-Aldrich Merck, Missouri, USA), and detected by fluorescence spectrophotometer at 340 nm. The histamine releases of basophils which pre-sensitized with serum from non-allergic healthy individual triggered by mite or shrimp were used as a negative control. The spontaneous release of histamine is measured in basophils by incubating at 37 °C for 60 minutes. The stimulation in the release of histamine is, indeed, the histamine content of basophils induced by shrimp or mite. The total released histamine was measured by treating basophils in PMNs with calcium ionophore A23187 (10 μmol/l) at 37 °C for 60 minutes. The following formula calculated the percentage of histamine release: (stimulated released histamine – spontaneously released histamine)/total released histamine × 100%.

### Cross-reactivity assay

For the inhibition of histamine release assay, serum samples were selected from 10 un-adults, 15 adults, and 11 elderly allergic subjects who had sIgE levels for mite and shrimp allergies greater than 3.5 kUA/L. Each serum sample was pre-absorbed with 50 μg/ml of recombinant Pen a 1 (rPen a 1) or Der p 10 (rDer p 10) and left overnight at 4 °C. After pre-absorption, the subsequent procedures for detecting the release of histamine using the BHR assay were the same as those described above. The inhibition of histamine release was calculated by following formula: (the percentage of allergen triggered histamine release – the percentage of allergen triggered histamine release with pre-absorption)/the percentage of allergen triggered histamine release × 100%.

### Immunoblot inhibition assay of IgE in vegetarians

Four vegetarians in our cohort were selected who did not report eating meat, including seafood, at least in the last seven years. *D. pteronyssinus* crude extract was separated on a gel and then transferred onto a polyvinylidene difluoride (PVDF) membrane. The membranes were blocked with 3% (w/v) skimmed milk in phosphate buffered saline with 1% Tween-20 (PBST). The blots were incubated with 100 μl diluted serum samples (5-fold dilution in PBST) from four vegetarians (V1~V4) at 4 °C for 16 h. The membranes were then incubated with horseradish peroxidase (HRP)-conjugated monoclonal anti-human IgE antibodies (1:1,000 dilution), (Sigma-Aldrich Merck, Missouri, USA) at 25 °C for 2 h. Membranes were washed with PBST three times. Finally, the blots were developed for signal detection with ECL reagent (Bio-Rad, California, USA), and were recorded photographically.

### Statistical analysis

Data regarding different age groups, species, and allergens were represented as the mean ± standard deviation (SD) for each group. The results of sIgE level of mite and shrimp in the three age groups were analysed by analysis of variance (ANOVA) with Tukey’s multiple comparisons post-test. The p-values less than 0.05 were considered statistically significant. The differences between two parameters were analysed with a non-paired t-test.

## Supplementary information

Supplementary Figures

## Data Availability

All data generated or analysed during this study are included in this published article (and its supplementary information files).
