# Supplementary material for: Cross-reactivity of sIgE to mite and shrimp induced allergies in different age groups and clinical profiles of shrimp sIgE in vegetarians
Source: Sci Rep. 2019 Aug 29;9:12548. doi: 10.1038/s41598-019-49068-2 (PMC6715687; doi:10.1038/s41598-019-49068-2)
Supplement: Supplementary file 1 — Supplementary Figures [file 41598_2019_49068_MOESM1_ESM.doc]

Cross-reactivity of sIgE to mite and shrimp induced allergies in different age groups and clinical profiles of shrimp sIgE in vegetarians

*Cheng-Ying Shen^1^*^,#^*, Jaw-Ji Tsai^2,3,4^,* ^#^*,* *En-Chih Liao^1,*^*

**^1^** Department of Medicine, Mackay Medical College, New Taipei City, Taiwan, R.O.C.

**^2^** Department of Medical Research, Taichung Veterans General Hospital, Taiwan, R.O.C.

**^3^** Institute of Clinical Medicine, National Yang Ming University, Taipei, Taiwan, R.O.C.

**^4^** College of Life Sciences, National Chung Hsing University, Taichung, Taiwan, R.O.C.

#These authors contributed equally to the supervision of this work

***Corresponding Author:**

Dr. En-Chih Liao. PhD.

Department of Medicine, Mackay Medical College No. 46, Sec. 3, Zhongzheng Rd., Sanzhi Dist., New Taipei City 252, Taiwan

Tel：+886-2-26360303 # 1244

E-mail address: enchih@mmc.edu.tw (E.-C. Liao)

**
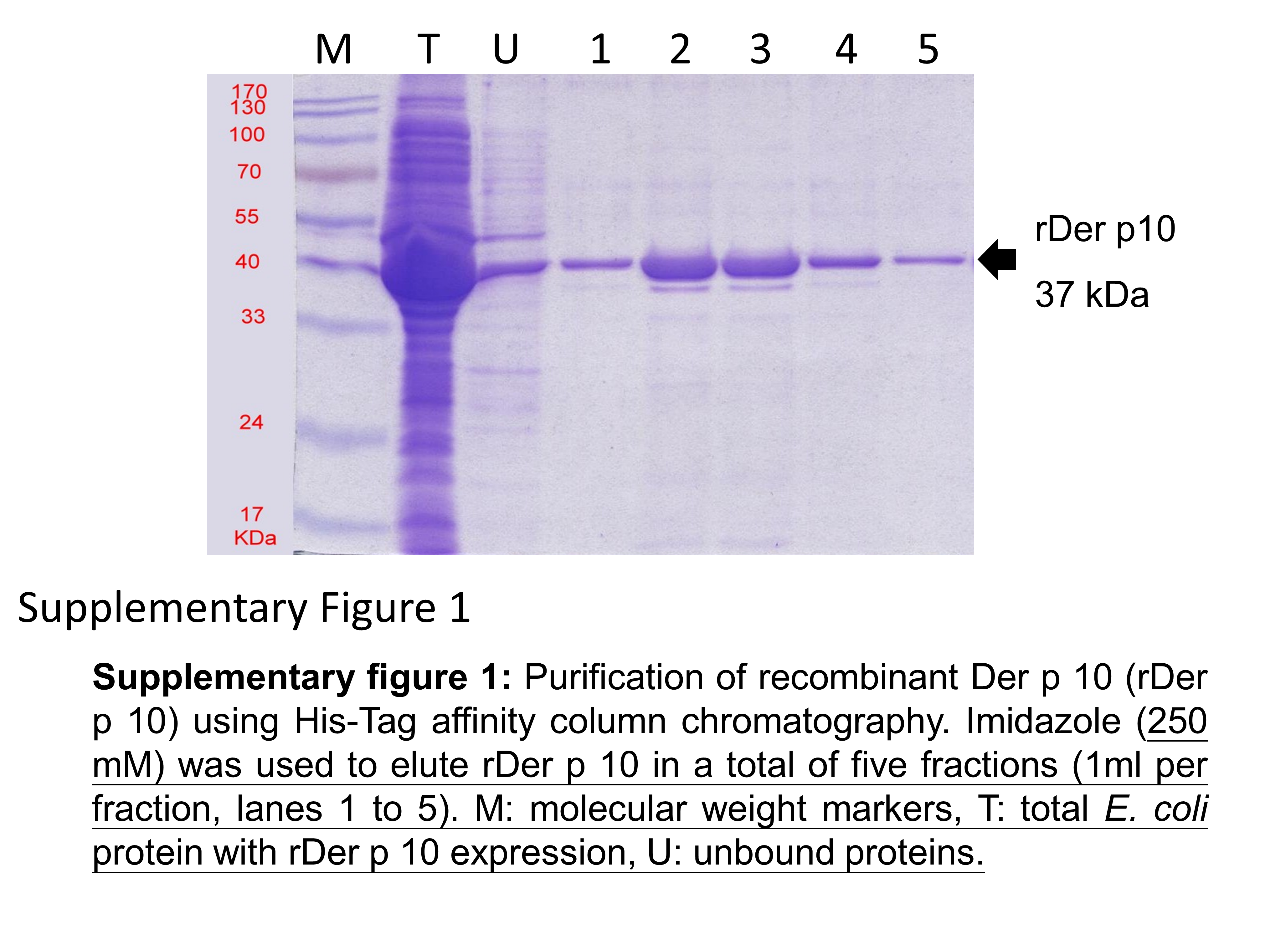
**

**Supplementary figure 1:** Purification of recombinant Der p 10 (rDer p 10) using His-Tag affinity column chromatography. Imidazole (250 mM) was used to elute rDer p 10 in a total of five fractions (1ml per fraction, lanes 1 to 5). M: molecular weight markers, T: total *E. coli* protein with rDer p 10 expression, U: unbound proteins.

**
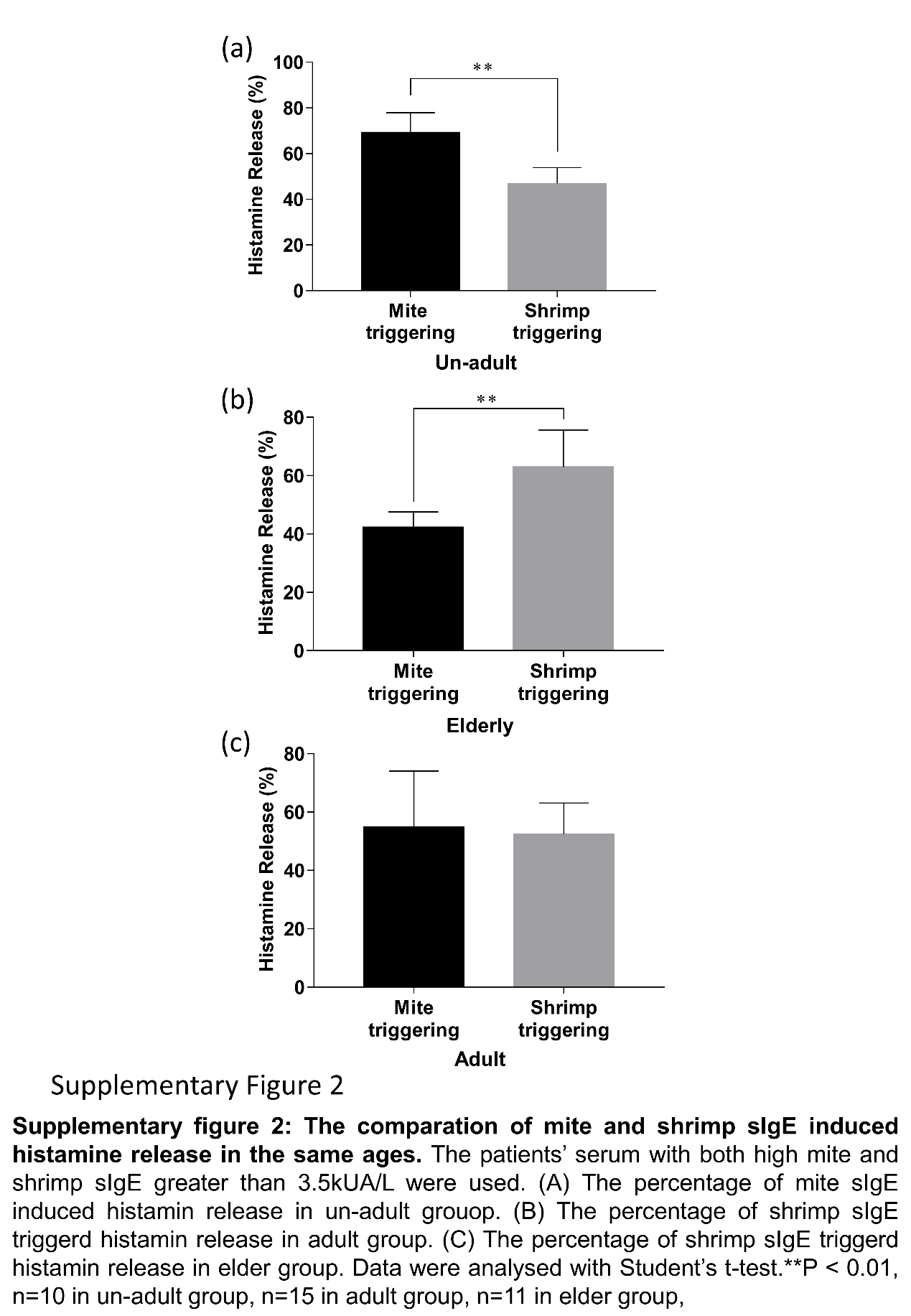
**

**Supplementary figure 2: The comparation of mite and shrimp sIgE induced histamine release in the same ages.** The patients’ serum with both high mite and shrimp sIgE greater than 3.5kUA/L were used. (A) The percentage of mite sIgE induced histamine release in un-adult group. (B) The percentage of shrimp sIgE triggered histamine release in elder group. (C) The percentage of shrimp sIgE triggered histamine release in adult group. Data were analyzed with Student’s t-test.**P < 0.01, n=10 in un-adult group, n=11 in elder group, n=15 in adult group.


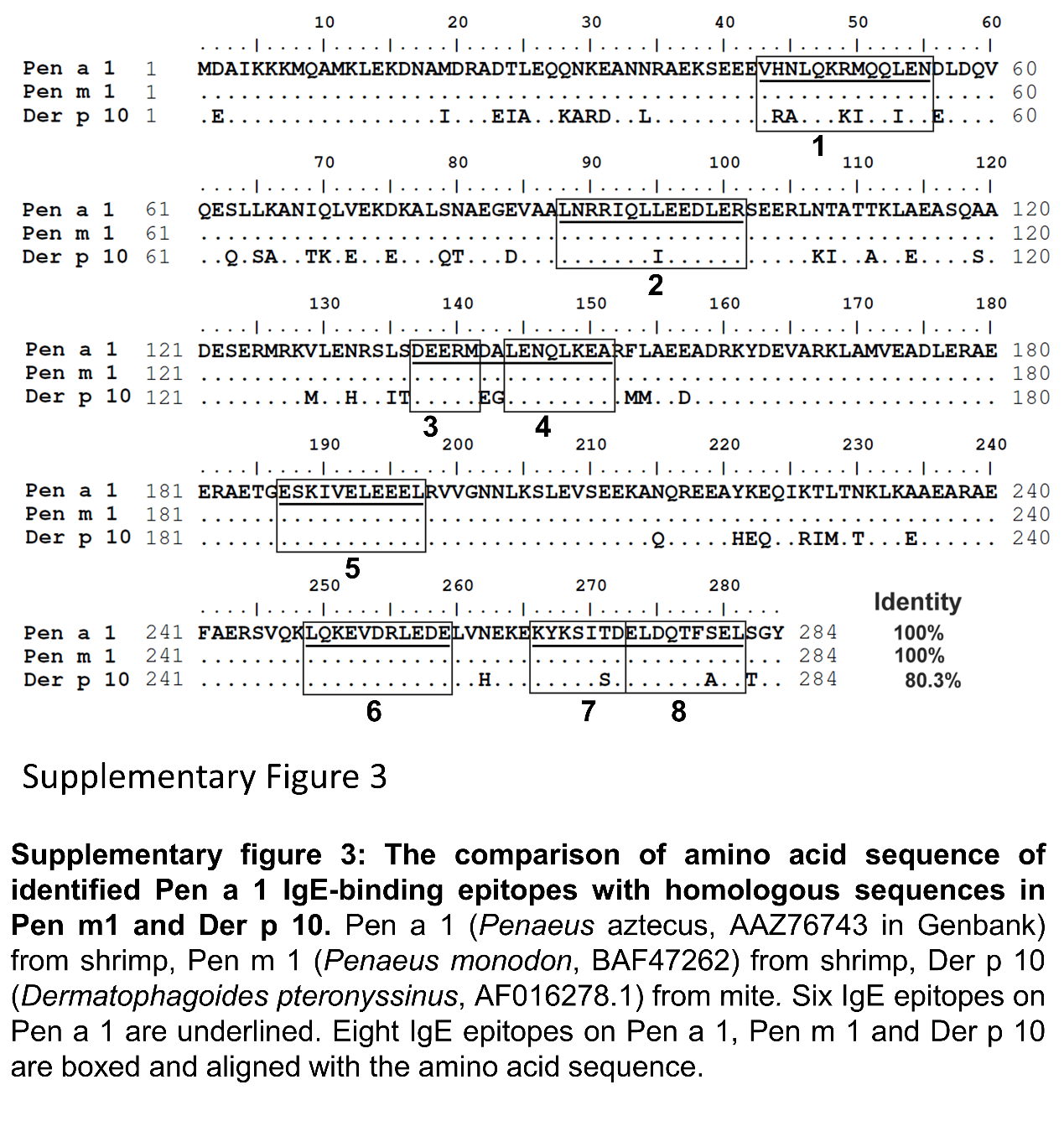


**Supplementary figure 3: The comparison of amino acid sequence of identified Pen a 1 IgE-binding epitopes with homologous sequences in Pen m1 and Der p 10.** Pen a 1 (*Penaeus* aztecus, AAZ76743 in Genbank) from shrimp, Pen m 1 (*Penaeus monodon*, BAF47262) from shrimp, Der p 10 (*Dermatophagoides pteronyssinus*, AF016278.1) from mite*.* Six IgE epitopes on Pen a 1 are underlined. Eight IgE epitopes on Pen a 1, Pen m 1 and Der p 10 are boxed and aligned with the amino acid sequence.
